# Association Between CT‐Based Rectal Stool Attenuation and Activities of Daily Living in Hospitalized Older Adults

**DOI:** 10.1111/ggi.70867

**Published:** 2026-10-13

**Authors:** Hitoshi Komiya, Kazuhisa Watanabe, Masaaki Nagae, Hirotaka Nakashima, Chisato Fujisawa, Tomihiko Tajima, Yosuke Yamada, Hiroyuki Umegaki

**Affiliations:** ^1^ Centre for Community Liaison and Patient Consultations Nagoya University Hospital Nagoya Aichi Japan; ^2^ Department of Community Healthcare and Geriatrics Nagoya University Graduate School of Medicine Nagoya Aichi Japan; ^3^ Institute of Innovation for Future Society Nagoya University Nagoya Aichi Japan

**Keywords:** activities of daily living, computed tomography, geriatrics, rectum, stool attenuation

## Abstract

**Objectives:**

To explore the association between rectal stool characteristics measured by computed tomography (CT), particularly stool attenuation, and activities of daily living (ADL) in hospitalized older adults who had previously undergone abdominal CT for clinical reasons.

**Methods:**

This single‐center exploratory cross‐sectional study included adults aged ≥ 65 years admitted to the geriatric ward of Nagoya University Hospital who underwent abdominal CT within 1 month before or during hospitalization. Basic ADL (BADL) and instrumental ADL (IADL) were assessed using the Barthel Index and the Lawton and Brody IADL scale, respectively. Rectal stool attenuation, rectal lumen area, stool area, and gas area were measured on the axial slice with the largest rectal cross‐section. High‐attenuation stool was defined as > 100 HU, and 10 cm^2^ was used as the threshold for rectal area parameters. Correlations between continuous CT parameters were examined using Spearman's rank correlation. Associations between CT findings and ADL were first examined by comparing mean ADL scores between groups defined by CT parameters; multivariable logistic regression was then used to evaluate factors independently associated with high‐attenuation stool. Odds ratios (ORs) and 95% confidence intervals (CIs) were calculated.

**Results:**

Among 116 participants (mean age, 85.8 years), 31.0% had high‐attenuation stool. High‐attenuation stool was associated with significantly lower BADL scores (55.4 ± 38.0 vs. 74.4 ± 27.5) and IADL scores (2.4 ± 2.9 vs. 3.9 ± 2.8). Stool attenuation was positively correlated with stool area (*r* = 0.34) and negatively correlated with gas area (*r* = −0.31). In multivariable analysis, lower BADL (OR, 0.983; 95% CI, 0.970–0.997) and lower IADL (OR, 0.840; 95% CI, 0.715–0.986) were independently associated with high‐attenuation stool. When analyzed as a continuous variable, stool attenuation was negatively correlated with both BADL (*r* = −0.224, *p* = 0.015) and IADL (*r* = −0.199, *p* = 0.032).

**Conclusion:**

High rectal stool attenuation on CT was significantly associated with lower ADL in hospitalized older adults. These exploratory findings suggest that CT‐based rectal stool attenuation may provide supplementary imaging information regarding functional vulnerability in older adults who have previously undergone abdominal CT. However, the cross‐sectional design, CT‐selected study population, and lack of contemporaneous assessment of constipation symptoms, bowel habits, or stool form preclude conclusions regarding causality or clinical utility.

## Graphical abstract

Higher rectal stool attenuation on abdominal CT was associated with lower BADL and IADL scores. This parameter may offer additional functional information from CT examinations already obtained for clinical indications.

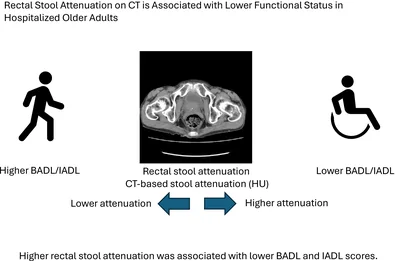

## 1 Introduction

Constipation is a multifaceted condition characterized by symptoms such as pellet‐like or hard stools and reduced bowel movement frequency due to fecal retention [1, 2, 3]. It also includes defecation‐related symptoms, such as excessive straining, a sense of incomplete evacuation, a feeling of anorectal blockage, and difficulty passing stool.

Several studies have reported examinations of the relationship between CT‐based assessments of fecal retention and constipation symptoms [4, 5]. Although colonic stool burden on CT does not necessarily correlate with clinical constipation [6], rectal gas accumulation has been associated with evacuation disorders [7] and rectal stool attenuation on CT has been shown to correlate with stool form assessed using the Bristol Stool Form Scale (BSFS) [8].

Among older adults, decreased activities of daily living (ADL) have been reported as a risk factor for chronic constipation [9, 10, 11, 12]. Reduced physical activity may impair bowel motility, thereby contributing to constipation [13, 14, 15]. However, despite evidence linking CT findings with constipation symptoms and chronic constipation with decreased ADL in older adults, no previous studies have directly reported the association between CT‐based rectal stool characteristics and ADL in this population.

Therefore, in this exploratory study, we aimed to evaluate the association between CT‐based rectal stool findings and ADL in hospitalized older adults. We specifically examined whether CT‐based stool attenuation, an imaging characteristic that has been associated with stool hardness, was more strongly associated with ADL than rectal stool area, rectal gas area, or rectal distension. The purpose of this study was not to diagnose clinical constipation using CT, but rather to determine whether rectal stool attenuation observed on abdominal CT previously obtained for routine clinical care is associated with functional status in hospitalized older adults. If such an association exists, CT‐based stool attenuation may provide supplementary objective information in patients who have difficulty communicating their bowel symptoms.

## 2 Methods

### 2.1 Study Setting and Participants

In this cross‐sectional study, we used data from a prospective cohort of acutely hospitalized older adults. Among all patients admitted to the geriatric ward of Nagoya University Hospital between October 2019 and September 2020, those who met any of the following exclusion criteria were excluded: (i) discharge within 48 h; (ii) lack of written informed consent from the patient or a family member; (iii) life expectancy at admission, as estimated by the attending physician, of less than 1 month; (iv) readmission within 3 months of discharge after enrollment during a previous admission; and (v) transfer from another department. The remaining patients were enrolled in the cohort.

For the present analysis, we retrospectively identified and included only enrolled patients who underwent abdominal CT within 30 days before admission or during hospitalization and whose CT images were free of artifacts caused by artificial hip joints or other metallic devices.

Abdominal CT examinations were performed for routine clinical indications at the discretion of the treating physicians and were not obtained specifically for research purposes or for the assessment of bowel status.

### 2.2 Data Collection and Clinical Assessments

Background information, including age, sex, height, weight, and body mass index, was obtained from clinical records. The attending geriatrician conducted a comprehensive geriatric assessment (CGA) to evaluate functional status, cognition, nutritional status, and depressive symptoms. Baseline functional status (2 weeks prior to admission) was assessed using the Eastern Cooperative Oncology Group Performance Status (PS), which has been adopted by the World Health Organization [16]. Basic ADL (BADL) (2 weeks prior to admission) were assessed using the Barthel Index (BI) [17]. Instrumental ADL (IADL) (2 weeks prior to admission) were assessed using the Lawton and Brody scale [18]. Nutritional status (within 2 days of admission) was evaluated using the Mini Nutritional Assessment–Short Form (MNA‐SF) [19]. Cognitive function (within 2 days of admission) was assessed using the Mini‐Mental State Examination (MMSE) [20]. Depressive symptoms (within 2 days of admission) were assessed using the 15‐item Geriatric Depression Scale (GDS‐15) [21]. Comorbidity burden (within 2 days of admission) was evaluated using the Charlson Comorbidity Index (CCI) [22]. Frailty (2 weeks prior to admission) was assessed by a single physician using the Japanese version of the Clinical Frailty Scale (CFS‐J) [23, 24]. A timeline illustrating the temporal relationships among CT acquisition, ADL assessment, and other clinical assessments is shown in Figure 1.

**FIGURE 1:**
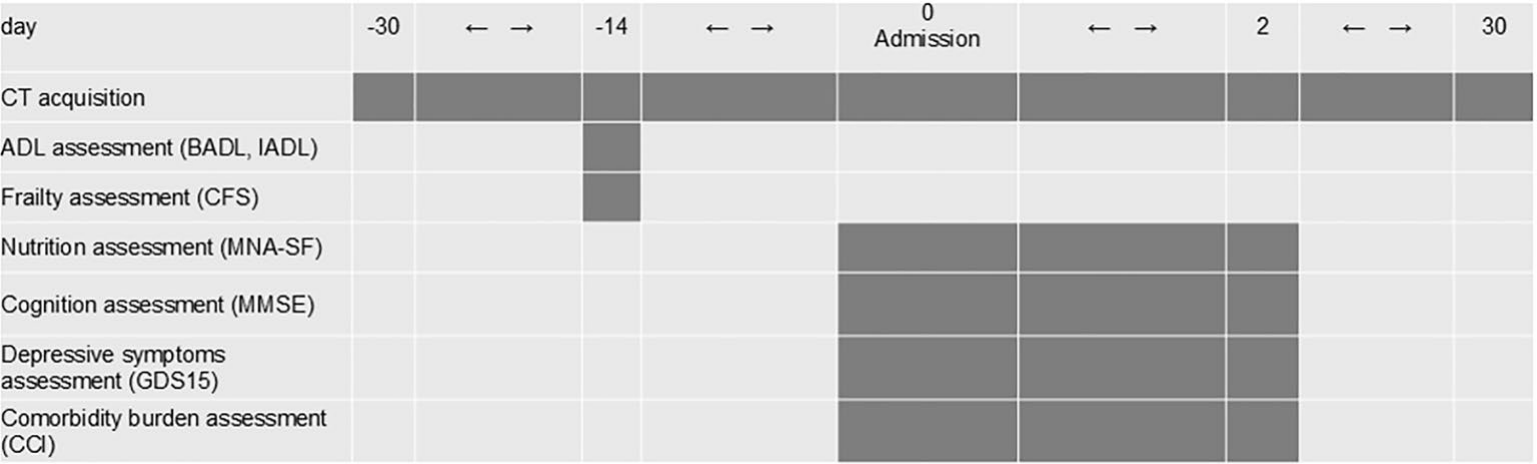
Timeline of clinical assessment. Abdominal CT was performed within 30 days before admission or during hospitalization. BADL, IADL, and CFS were assessed based on participants' status 2 weeks before admission. Nutritional status (MNA‐SF), cognitive function (MMSE), depressive symptoms (GDS‐15), and comorbidity burden (CCI) were assessed within 2 days after admission.

### 2.3 Definition and Measurement of CT‐Based Rectal Parameters

Continuous variables were summarized as median and interquartile range (IQR), or mean and standard deviation (SD), as appropriate. The vertical range for evaluating the rectal lumen, stool, and gas was standardized from the axial slice at the inferior margin of the pubic symphysis to the slice marking the entrance to the lower pelvis, corresponding to the inferior margin of the sacroiliac joints [25]. On the axial slice with the largest rectal lumen, we measured stool attenuation, the cross‐sectional area of the rectal lumen, the area of stool within the lumen, and the area of gas within the lumen.

Stool attenuation was assessed by placing a region of interest (ROI) within the rectal lumen. Using SYNAPSE SAI Viewer software (Fujifilm Medical, Tokyo, Japan), the maximum attenuation value (HU) within the ROI was automatically calculated and recorded for analysis.

The rectal cross‐sectional area was calculated using the same software by measuring the longest and shortest diameters and applying an elliptical approximation (1/4 × π × long diameter × short diameter). The rectal gas area was obtained from lung‐window images by manually tracing the gas‐containing region. The rectal stool area was calculated as the rectal cross‐sectional area minus the rectal gas area.

These parameters were defined as the maximum CT attenuation value of rectal stool, maximum rectal luminal area on CT, maximum rectal stool area on CT, and maximum rectal gas area on CT, respectively.

Based on previous imaging studies reporting that higher CT attenuation reflects harder stool, a threshold of > 100 HU was used to define high‐attenuation stool [8]. In addition, based on a previous report that proposed a rectal gas volume threshold for screening evacuation disorders, a cut‐off value of 10 cm^2^ was adopted for rectal gas area in the present study [26]. The same 10 cm^2^ threshold was also applied to the maximum rectal cross‐sectional area and rectal stool area for exploratory comparison across rectal parameters. All measurements related to rectal stool characteristics and areas were performed by a single trained physician. CT measurements were performed using anonymized CT images, and the evaluator did not review ADL data during the measurement process. The number of days from admission (day 0) to CT acquisition had a median (IQR) of 0 (0, 0), with a mean ± SD of 0.6 ± 3.3 days. The distribution of the interval between admission and CT acquisition is shown in Figure S1. CT scans performed before admission were assigned negative values.

### 2.4 Statistical Analysis

Descriptive statistics are presented as *n* (%) for categorical variables and as mean ± standard deviation and median with first and third quartiles for continuous variables. Spearman's rank correlation coefficient was used to examine correlations between continuous CT parameters. Participants were divided into two groups according to CT‐based rectal parameters: high‐ versus low‐attenuation stool and ≥ 10 cm^2^ versus < 10 cm^2^ for rectal area parameters. Mean ADL scores were compared between groups using Student's or Welch's *t*‐test, as appropriate. Missing data were handled using complete‐case analysis, and the number of missing observations for each variable is reported in Table 1. Multivariable logistic regression analysis was conducted to examine whether ADL measures were independently associated with high‐attenuation stool after adjustment for clinically relevant covariates. BADL and IADL were entered into separate multivariable models because they represent related but distinct aspects of functional status and may be correlated, potentially resulting in multicollinearity if included in the same model. As covariates in the multivariable logistic regression model, we included age, sex, CCI, Clinical Frailty Scale (CFS), and laxative use, as these factors have been suggested to be related to constipation. To assess intra‐rater reliability, 30 randomly selected CT scans were re‐evaluated by the same observer more than 3 months after the initial assessment. Intraclass correlation coefficients (ICCs) were calculated using a two‐way mixed‐effects model with absolute agreement for CT attenuation, rectal area, stool area, and gas area measurements. All analyses were conducted using SPSS version 31 (IBM Corp., Armonk, NY, USA), and two‐sided *p*‐values < 0.05 were considered statistically significant.

**TABLE 1:** Baseline characteristics of the study participants at admission.

	October 2019–September 2020	Missing data
Number of participants	116	0
Female sex, *n* (%)	69 (59.5%)	0
Age, years, mean (SD); median (Q1, Q3)	85.8 (5.9); 86 (82, 89)	0
BMI, kg/m^2^	20.3 (4.1); 19.8 (17.5, 22.7)	9
Maximum CT attenuation value of rectal stool, HU	95.8 (88.8); 74 (59.3, 108.8)	0
Maximum CT attenuation value of rectal stool > 100 HU, *n* (%)	36 (31.0%)	0
Maximum rectal cross‐sectional area, cm^2^	15.3 (10.7), 12.9 (7.4, 20.2)	0
Maximum rectal cross‐sectional area > 10 cm^2^, *n* (%)	71 (61.2%)	0
Rectal stool area at the maximum cross‐section, cm^2^	11.5 (10.0), 8.4 (4.4, 16.1)	0
Rectal stool area at the maximum cross‐section > 10 cm^2^, *n* (%)	51 (44.0%)	0
Rectal gas area at the maximum cross‐section, cm^2^	3.8 (5.5), 1.6 (0.2, 5.1)	0
Rectal gas area at the maximum cross‐section > 10 cm^2^, *n* (%)	13 (11.2%)	0
CCI score	2.7 (2.2), 2 (1, 4)	0
GDS score	5.2 (3.7), 4 (2, 8)	32
MMSE score	15.9 (9.3), 18 (8, 23)	5
MNA‐SF score	7.9 (3.4), 8 (5, 11)	11
PS score	1.9 (1.3), 2 (1, 3)	2
CFS score	5.6 (1.5), 6 (5, 7)	4
BI score	68.5 (32.2), 80 (50, 95)	0
IADL score	3.4 (2.9), 3 (0, 6)	0
Number of prescribed medications	6.7 (4.2), 6 (4, 10)	0
Number of PIMs	1.7 (1.6), 1 (1, 2)	0
ACRS score	1.3 (1.4), 1 (0, 2)	0
Laxative use, *n* (%)	39 (33.6%)	0
Stimulant laxative use, *n* (%)	13 (11.1%)	0
Non‐stimulant laxative use, *n* (%)	28 (24.1%)	0

*Note:* Values are presented as mean (SD); median (Q1, Q3) unless otherwise indicated.

Abbreviations: ACRS, anticholinergic risk scale; BI, Barthel Index; BMI, body mass index; CCI, Charlson Comorbidity Index; CFS, Clinical Frailty Scale; GDS, Geriatric Depression Scale; HU, Hounsfield units; IADL, instrumental activities of daily living; MMSE, Mini‐Mental State Examination; MNA‐SF, Mini Nutritional Assessment–Short Form; PIM, potentially inappropriate medication; PS, Performance Status; Q1, first quartile; Q3, third quartile; SD, standard deviation.

## 3 Results

### 3.1 Study Population Characteristics

A total of 156 inpatients met the initial eligibility criteria after exclusion criteria 1–5 were applied. Of these 156 patients, 29 had not undergone abdominal CT during the study period, and 11 were excluded because abdominal CT attenuation values near the rectum could not be accurately evaluated owing to artificial implants or other factors. Therefore, 116 patients were included in the final analysis. Among the 116 participants, the mean age was 85.8 ± 5.9 years, 69 (59.5%) were women, and the mean body mass index was 20.3 ± 4.1 kg/m^2^.

The mean maximum CT attenuation value of rectal stool, maximum rectal cross‐sectional area, maximum rectal cross‐sectional stool area, and maximum rectal cross‐sectional gas area were 95.8 ± 88.8 HU, 15.3 ± 10.7 cm^2^, 11.5 ± 10.0 cm^2^, and 3.8 ± 5.5 cm^2^, respectively. The numbers of participants with a maximum CT attenuation value of rectal stool > 100 HU, maximum rectal cross‐sectional area > 10 cm^2^, maximum rectal cross‐sectional stool area > 10 cm^2^, and maximum rectal cross‐sectional gas area > 10 cm^2^ were 36 (31.0%), 71 (61.2%), 51 (44.0%), and 13 (11.2%), respectively.

Additional participant characteristics are presented in Table 1.

### 3.2 Comparison of BADL and IADL According to CT‐Based Rectal Parameters

When participants were divided into the high‐attenuation stool group (> 100 HU; *n* = 36) and low‐attenuation stool group (≤ 100 HU; *n* = 80), mean BADL scores (55.4 ± 38.0 vs. 74.4 ± 27.5) and IADL scores (2.4 ± 2.9 vs. 3.9 ± 2.8) were significantly lower in the high‐attenuation stool group (Table 2).

**TABLE 2:** Comparison of BADL and IADL according to CT‐based high‐attenuation stool versus low‐attenuation stool.

	High‐attenuation stool (> 100 HU; *n* = 36)	Low‐attenuation stool (≤ 100 HU; *n* = 80)	*p*
BADL score, mean (SD)	55.4 (38.0)	74.4 (27.5)	0.010
IADL score, mean (SD)	2.4 (2.9)	3.9 (2.8)	0.010

Abbreviations: BADL, basic activities of daily living; CT, computed tomography; HU, Hounsfield units; IADL, instrumental activities of daily living; SD, standard deviation.

In contrast, no significant differences in mean BADL or IADL scores were observed between the large and small rectal area groups (> 10 cm^2^ vs. ≤ 10 cm^2^), large and small rectal stool area groups (> 10 cm^2^ vs. ≤ 10 cm^2^), or large and small rectal gas area groups (> 10 cm^2^ vs. ≤ 10 cm^2^) (Table S1).

### 3.3 Relationship Between Rectal Stool Attenuation and Rectal Cross‐Sectional Measures

The maximum CT attenuation value of rectal stool was positively correlated with rectal stool area (*r* = 0.342, *p* < 0.001) and negatively correlated with rectal gas area (*r* = −0.312, *p* < 0.001), whereas no significant correlation was observed with maximum rectal cross‐sectional area (*r* = 0.164, *p* = 0.078). These results are presented in Table 3.

**TABLE 3:** Correlations between maximum CT attenuation value of rectal stool and CT‐based rectal area parameters.

	Maximum rectal cross‐sectional area		Rectal stool area at the maximum cross‐section		Rectal gas area at the maximum cross‐section	
	*r*	*p*	*r*	*p*	*r*	*p*
Maximum CT attenuation value of rectal stool	0.164	0.078	0.342	*p* < 0.001	−0.312	*p* < 0.001

Abbreviations: CT, computed tomography; *r*, Spearman's rank correlation coefficient.

### 3.4 Comparison of Individual BADL Items Between the High‐ and Low‐Attenuation Stool Groups

As an exploratory analysis, individual BADL item scores were compared between the high‐attenuation stool (> 100 HU) and low‐attenuation stool (≤ 100 HU) groups. The high‐attenuation stool group had lower scores for feeding (7.1 ± 4.2 vs. 8.8 ± 2.5), dressing (5.3 ± 4.6 vs. 7.6 ± 3.6), bowels (5.3 ± 4.3 vs. 7.4 ± 3.6), bladder (4.9 ± 3.9 vs. 7.1 ± 3.6), toilet use (5.4 ± 4.5 vs. 7.9 ± 3.3), transfer (9.9 ± 5.4 vs. 12.7 ± 3.7), and stairs (4.0 ± 4.1 vs. 5.9 ± 4.2). In contrast, no significant differences were observed for bathing (2.1 ± 2.5 vs. 2.5 ± 2.5), grooming (2.8 ± 2.5 vs. 3.6 ± 2.3), or mobility (8.8 ± 6.5 vs. 10.8 ± 5.3). These exploratory item‐level comparisons are presented in Table 4.

**TABLE 4:** Comparison of individual BADL items between the high‐ and low‐attenuation stool groups.

BADL item	High‐attenuation stool (> 100 HU), mean ± SD	Low‐attenuation stool (≤ 100 HU), mean ± SD	*p*
Feeding	7.1 ± 4.2	8.8 ± 2.5	0.027
Bathing	2.1 ± 2.5	2.5 ± 2.5	0.410
Grooming	2.8 ± 2.5	3.6 ± 2.3	0.115
Dressing	5.3 ± 4.6	7.6 ± 3.6	0.009
Bowels	5.3 ± 4.3	7.4 ± 3.6	0.005
Bladder	4.9 ± 3.9	7.1 ± 3.6	0.003
Toilet use	5.4 ± 4.5	7.9 ± 3.3	0.005
Transfer	9.9 ± 5.4	12.7 ± 3.7	0.006
Mobility	8.8 ± 6.5	10.8 ± 5.3	0.074
Stairs	4.0 ± 4.1	5.9 ± 4.2	0.025

*Note: p*‐values were calculated using Student's or Welch's *t*‐test, as appropriate.

Abbreviations: BADL, basic activities of daily living; HU, Hounsfield units; SD, standard deviation.

### 3.5 Logistic Regression Analysis of Factors Associated With High‐Attenuation Stool

Table 5 shows the results of the multivariable analysis, in which high‐attenuation stool was used as the dependent variable. BADL and IADL were entered as explanatory variables in separate models, with age, sex, CCI, CFS, and laxative use included as covariates. Lower BADL (OR = 0.983, 95% CI 0.970–0.997, *p* = 0.015) and lower IADL (OR = 0.840, 95% CI 0.715–0.986, *p* = 0.033) were independently associated with high‐attenuation stool.

**TABLE 5:** Logistic regression analysis of factors associated with high‐attenuation stool.

Variable	OR	95% CI	*p*	OR	95% CI	*p*
Age	0.953	0.886–1.025	0.194	0.965	0.898–1.037	0.332
Female sex	1.360	0.554–3.336	0.502	1.475	0.598–3.635	0.399
CCI	0.955	0.783–1.164	0.647	0.939	0.771–1.145	0.536
CFS	1.053	0.760–1.459	0.756	1.042	0.747–1.453	0.810
Laxative use	0.846	0.336–2.131	0.760	0.837	0.363–2.271	0.837
BADL score (BI)	0.983	0.970–0.997	0.015			
IADL score (Lawton and Brody scale)				0.840	0.715–0.986	0.033

*Note:* Reference categories were male (gender) and no laxative use (laxative use). Age, CCI, CFS, BADL score, and IADL score were treated as continuous variables. Model 1 included BADL score; Model 2 included IADL score. Both models were adjusted for age, female sex, CCI, CFS, and laxative use.

Abbreviations: BADL, basic activities of daily living; BI, Barthel Index; CCI, Charlson Comorbidity Index; CFS, clinical frailty scale; CI, confidence interval; IADL, instrumental activities of daily living; OR, odds ratio.

### 3.6 Intra‐Rater Reliability of CT Measurements

To assess intra‐rater reliability, 30 randomly selected CT scans were re‐evaluated by the same observer more than 3 months after the initial assessment. CT attenuation measurements demonstrated excellent reliability, with an ICC of 0.958 (95% CI, 0.914–0.979). Rectal area measurements also showed excellent reliability (ICC, 0.935; 95% CI, 0.863–0.969). Stool area measurements further evidenced excellent reliability (ICC, 0.961; 95% CI, 0.920–0.981), whereas gas area measurements showed moderate reliability (ICC, 0.724; 95% CI, 0.503–0.857). These results are shown in Table S2.

### 3.7 Association Between Stool Attenuation and Functional Status

To assess whether the observed associations were dependent on the predefined cutoff value of 100 HU, additional analyses were performed using stool attenuation as a continuous variable. Spearman's rank correlation analysis showed that stool attenuation was negatively correlated with both BADL (*r* = −0.224, *p* = 0.015) and IADL (*r* = −0.199, *p* = 0.032). These findings indicate that higher stool attenuation was associated with poorer functional status even when attenuation was analyzed as a continuous measure.

## 4 Discussion

### 4.1 Summary of Findings

In this exploratory study of hospitalized older adults who had previously undergone abdominal CT for clinical reasons, high‐attenuation stool in the rectum was significantly associated with lower ADL. In contrast, no significant associations were observed between ADL and rectal size, stool area, or gas area. The maximum CT attenuation value of rectal stool was positively correlated with rectal stool area at the maximum cross‐section and negatively correlated with rectal gas area at the maximum cross‐section. Because rectal cross‐sectional area is the sum of stool and gas areas, correlations among these three measures are mathematically interdependent; therefore, we focused on correlations between stool attenuation and each component, namely stool area and gas area, rather than examining correlations among all three measures. Individuals in the high‐attenuation stool group also had lower mean scores in several ADL items, including feeding, dressing, bowels, bladder, toilet use, transfer, and stairs. These item‐level findings should be interpreted as exploratory because multiple comparisons were performed. In multivariable analyses, lower BADL and IADL scores remained independently associated with high‐attenuation stool after adjustment for prespecified covariates. Taken together, these findings suggest that CT‐based rectal stool attenuation may provide supplementary imaging information regarding functional vulnerability in older adults who have previously undergone abdominal CT, although the cross‐sectional design precludes conclusions regarding causality or directionality.

### 4.2 Comparison With Previous Studies

Previous studies have reported that constipation in older adults is associated with reduced intestinal motility, impaired pelvic floor function, and lower ADL [14, 15]. In addition, correlations have been reported between stool consistency, as assessed using the BSFS, and CT attenuation values of rectal stool, as well as between chronic constipation symptoms and CT‐based rectal stool characteristics [7, 8]. However, few studies have reported the association between CT‐based stool attenuation and ADL, and in the present study, we address this gap.

Previous reports indicated that rectal volume and rectal stool volume were not significantly correlated with the BSFS, whereas rectal gas volume was negatively correlated with the BSFS [7]. In the present study, the CT attenuation value of rectal stool was positively correlated with rectal stool area and negatively correlated with rectal gas area. This discrepancy may be attributable to differences in the methods used to evaluate stool characteristics, specifically the use of the BSFS in earlier studies and maximum CT attenuation values in the present study. CT attenuation may reflect imaging characteristics related to stool water content, whereas the BSFS is a subjective, appearance‐based assessment.

Although CT attenuation has been reported to be associated with stool consistency, the extent to which it reflects clinically relevant constipation remains uncertain because clinical symptoms, bowel habits, and stool form were not assessed contemporaneously in the present study. Accordingly, our findings should be interpreted as demonstrating an association between CT‐based rectal stool attenuation and ADL, rather than between clinical constipation and ADL.

### 4.3 Potential Mechanisms Linking ADL Decline and High‐Attenuation Stool

In older adults, lower ADL has been reported as a risk factor for chronic constipation. Reduced physical activity may impair intestinal motility, which may in turn contribute to harder stools and constipation. In addition, decreased physical activity may weaken the pelvic floor muscles and reduce abdominal pressure, thereby contributing to defecation disorders. These mechanisms could potentially promote prolonged rectal stool retention and changes in stool characteristics [8, 15, 27].

The observed associations between lower basic and instrumental ADL and high‐attenuation stool are consistent with this proposed mechanism. Moreover, the lower scores for excretion‐related BADL items, such as bowels, bladder, and toilet use, in the high‐attenuation stool group may suggest a relationship between impaired toileting function and CT‐based rectal stool characteristics.

However, alternative explanations should also be considered. High‐attenuation stool may reflect factors associated with frailty, acute illness, dehydration, medication exposure, or bowel management practices rather than functional status alone. In addition, harder stool and impaired evacuation may themselves contribute to discomfort, reduced mobility, or further functional decline. Because of the cross‐sectional design, the temporal and causal relationships between ADL and CT‐based stool attenuation cannot be determined. Finally, the absence of associations between ADL and rectal size, stool area, or gas area suggests that stool attenuation may provide information distinct from simple measures of rectal stool burden. Although CT attenuation has been associated with stool consistency in previous studies, further research incorporating contemporaneous clinical assessments is needed to clarify its physiological and clinical significance.

### 4.4 Clinical Implications

Assessment of bowel status is often challenging in older adults with reduced ADL, cognitive impairment, or communication difficulties because symptom reporting may be unreliable or incomplete. In this context, imaging‐based assessment of rectal stool characteristics may provide supplementary objective information regarding bowel status and functional vulnerability.

Notably, the present findings should not be interpreted as supporting the use of CT as a screening tool for constipation, stool hardness, or functional decline. CT was performed for clinical indications unrelated to this study, and the potential value of CT‐based stool attenuation is limited to situations in which abdominal CT has previously been obtained as part of routine clinical care. In such circumstances, rectal stool attenuation may provide additional exploratory information beyond simple assessment of rectal stool volume or distension.

The potential significance of these findings may extend beyond CT itself. Because CT is associated with radiation exposure, cost, and limited feasibility for repeated assessment, it is not suitable for routine bowel evaluation in frail older adults. Rather, the present findings may support further investigation of noninvasive imaging‐based approaches for assessing rectal stool characteristics. For example, bedside ultrasonography may represent a practical and reproducible method for evaluating rectal stool retention and bowel status in older adults.

Our findings also suggest that stool attenuation, rather than the amount of stool retained in the rectum, may be a potentially informative imaging characteristic. However, CT‐based stool attenuation was not validated against contemporaneous constipation symptoms, BSFS scores, bowel movement frequency, or defecation difficulty. Therefore, these findings should be considered hypothesis‐generating and require confirmation in prospective studies incorporating imaging and clinical assessments.

### 4.5 Limitations

This study has some limitations. First, the cross‐sectional design precludes causal inference regarding the relationship between high‐attenuation rectal stool and lower ADL. Although reduced physical activity may plausibly contribute to impaired bowel motility and changes in stool characteristics, longitudinal studies are needed to clarify the directionality and temporal sequence of these associations.

Second, the timing of CT acquisition and clinical assessments was not fully aligned. Although most CT examinations were performed around the time of admission, ADL was assessed retrospectively based on functional status 2 weeks before admission, whereas cognitive and nutritional assessments were conducted within 2 days after admission. In addition, rectal stool characteristics were evaluated using a single CT scan obtained before or during acute hospitalization. Therefore, temporal mismatches among assessments may have influenced the observed associations.

Third, clinical measures of constipation, including bowel movement frequency, stool form, defecation difficulty, and other symptom‐based assessments, were not evaluated contemporaneously. Consequently, CT‐based rectal stool attenuation should not be interpreted as equivalent to clinical constipation.

Fourth, stool characteristics may fluctuate based on hydration status, dietary intake, medication use, bowel regimen, and acute illness. Although multiple covariates were included in the multivariable analyses, residual confounding due to unmeasured factors, such as fluid intake, dietary fiber intake, opioid use, anticholinergic medications, illness severity, and details of bowel management cannot be excluded.

Fifth, although CT‐based measurements were performed according to a standardized protocol and demonstrated high intra‐rater reliability, all measurements were obtained by a single trained physician, and inter‐rater reliability was not assessed. Therefore, the generalizability of the measurements to other evaluators remains uncertain.

Sixth, the number of patients with high‐attenuation stool was relatively small compared with the number of covariates included in the multivariable models. As a result, the regression estimates may be susceptible to overfitting and should be interpreted cautiously.

Define high‐attenuation stool (> 100 HU) and rectal area parameters were based on previous studies and may not be optimal for hospitalized older adults. Further studies are needed to validate these thresholds and to examine alternative approaches, including analyses using continuous variables.

Finally, the study was conducted in a single tertiary‐care geriatric ward and included only patients who underwent abdominal CT and had images suitable for rectal attenuation assessment. Therefore, selection bias is possible, and the findings may not be generalizable to broader older adult populations.

### 4.6 Conclusion

In this exploratory study of hospitalized older adults who had previously undergone abdominal CT for clinical reasons, high‐attenuation rectal stool was significantly associated with lower BADL and IADL. These findings suggest that CT‐based rectal stool attenuation may provide supplementary imaging information regarding functional vulnerability in this selected population. However, CT‐based stool attenuation was not validated against contemporaneous clinical measures of constipation, and the cross‐sectional design precludes conclusions regarding causality or clinical utility. Longitudinal studies incorporating constipation symptoms, stool‐form measures, bowel management data, and repeated clinical or imaging assessments are needed to further clarify the significance of this association.

## Author Contributions

**Hitoshi Komiya:** conceptualization, data curation, formal analysis, investigation, methodology, and writing – original draft. **Kazuhisa Watanabe:** project administration. **Masaaki Nagae:** project administration. **Yosuke Yamada:** resources and visualization. **Hirotaka Nakashima:** data curation, methodology, and validation. **Chisato Fujisawa:** investigation and writing – review and editing. **Tomihiko Tajima:** data curation. **Hiroyuki Umegaki:** writing – review and editing, supervision, and funding acquisition.

## Funding

This work was supported by the Japan Society for the Promotion of Science (JP21H02826).

## Ethics Statement

The study protocol was approved by the Ethics Committee of Nagoya University Graduate School of Medicine (approval number: 2019‐0260) and was conducted in accordance with the principles of the Declaration of Helsinki and its later amendments.

## Consent

Written informed consent was obtained from all study participants or their proxies.

## Conflicts of Interest

The authors declare no conflicts of interest.

## Supporting information


**Table S1:** Comparison of BADL and IADL According to other CT‐based Rectal Parameters.

**Table S2:** Intra‐rater reliability of CT‐based measurements.

**Figure S1:** Distribution of days from admission to CT acquisition. Histogram showing the distribution of the interval between hospital admission and CT acquisition among the study participants (*N* = 116). Negative values indicate CT examinations performed before admission, whereas positive values indicate CT examinations performed after admission. The mean interval was 0.57 days (SD, 3.33).

## Data Availability

The research data are not publicly available because consent for data sharing was not obtained from the participants.

## References

[1] F. Mearin , B. E. Lacy , L. Chang , et al., “Bowel Disorders,” Gastroenterology 150, no. 6 (2016): 1393–1407, 10.1053/j.gastro.2016.02.031. 27144627

[2] J. Serra , D. Pohl , F. Azpiroz , et al., “European Society of Neurogastroenterology and Motility Guidelines on Functional Constipation in Adults,” Neurogastroenterology and Motility 32, no. 2 (2020): e13762, 10.1111/nmo.13762. 31756783

[3] E. Ihara , N. Manabe , H. Ohkubo , et al., “Evidence‐Based Clinical Guidelines for Chronic Constipation 2023,” Digestion 106, no. 1 (2025): 62–89, 10.1159/000540912. PMC11825134 39159626

[4] H. Kawahara , N. Omura , and T. Akiba , “The Usefulness of Preoperative Evaluation for Intractable Slow Transit Constipation by Computed Tomography,” Journal of the Anus, Rectum and Colon 5, no. 2 (2021): 144–147, 10.23922/jarc.2020-065. PMC8084538 33937554

[5] T. Mizukami , S. Sugimoto , T. Masaoka , H. Suzuki , and T. Kanai , “Colonic Dysmotility and Morphological Abnormality Frequently Detected in Japanese Patients With Irritable Bowel Syndrome,” Intestinal Research 15, no. 2 (2017): 236–243, 10.5217/ir.2017.15.2.236. PMC5430017 28522955

[6] D. Ferguson , R. D. Holmes , R. Scott , et al., “Colonic Stool Burden on Computed Tomography Does Not Correlate With Bowel Habit: A Cross‐Sectional Study,” Abdominal Radiology 46, no. 2 (2021): 506–516, 10.1007/s00261-020-02689-y. 32761405

[7] M. Haraikawa , T. Takeda , S. Oki , et al., “Correlation Between Constipation Symptoms and Abdominal CT Imaging: A Cross‐Sectional Pilot Study,” Journal of Clinical Medicine 12, no. 1 (2023): 341, 10.3390/jcm12010341. PMC9820881 36615140

[8] K. Ishimaru , H. Kakuto , S. Yukumi , et al., “Imaging Classification of Constipation,” Journal of the Anus, Rectum and Colon 7, no. 1 (2023): 25–29, 10.23922/jarc.2022-039. PMC9876606 36743467

[9] M. Gustafsson , K. Lämås , U. Isaksson , P. O. Sandman , and H. Lövheim , “Constipation and Laxative Use Among People Living in Nursing Homes in 2007 and 2013,” BMC Geriatrics 19, no. 1 (2019): 38, 10.1186/s12877-019-1054-x. PMC6368797 30736737

[10] K. Lämås , S. Karlsson , A. Nolén , H. Lövheim , and P. O. Sandman , “Prevalence of Constipation Among Persons Living in Institutional Geriatric‐Care Settings: A Cross‐Sectional Study,” Scandinavian Journal of Caring Sciences 31, no. 1 (2017): 157–163, 10.1111/scs.12345. 27327073

[11] H. Komiya , H. Umegaki , A. Asai , et al., “Prevalence and Risk Factors of Constipation and Pollakisuria Among Older Home‐Care Patients,” Geriatrics & Gerontology International 19, no. 4 (2019): 277–281, 10.1111/ggi.13610. 30628140

[12] L. Alhalaseh , H. Makahleh , B. Al‐Saleem , F. Al‐Omran , and B. Schoenmakers , “Functional Status in Relation to Common Geriatric Syndromes and Sociodemographic Variables: A Step Forward Towards Healthy Aging,” Clinical Interventions in Aging 19 (2024): 901–910, 10.2147/CIA.S462347. PMC11110820 38779378

[13] P. Gallagher and D. O'Mahony , “Constipation in Old Age,” Best Practice & Research. Clinical Gastroenterology 23, no. 6 (2009): 875–887, 10.1016/j.bpg.2009.09.001. 19942165

[14] S. Mimura , A. Morishita , A. Kitaoka , et al., “Constipation in Older Adults: Pathophysiology, Clinical Impact, and Management Strategies,” Geriatrics 11, no. 2 (2026): 47, 10.3390/geriatrics11020047. PMC13116694 42042103

[15] B. Deb , D. O. Prichard , and A. E. Bharucha , “Constipation and Fecal Incontinence in the Elderly,” Current Gastroenterology Reports 22, no. 11 (2020): 54, 10.1007/s11894-020-00791-1. 32839874

[16] M. M. Oken , R. H. Creech , D. C. Tormey , et al., “Toxicity and Response Criteria of the Eastern Cooperative Oncology Group,” American Journal of Clinical Oncology 5, no. 6 (1982): 649–655. 7165009

[17] F. I. Mahoney and D. W. Barthel , “Functional Evaluation: The Barthel Index,” Maryland State Medical Journal 14 (1965): 61–65. 14258950

[18] M. P. Lawton and E. M. Brody , “Assessment of Older People: Self‐Maintaining and Instrumental Activities of Daily Living,” Gerontologist 9, no. 3 (1969): 179–186. 5349366

[19] L. Z. Rubenstein , J. O. Harker , A. Salvà , Y. Guigoz , and B. Vellas , “Screening for Undernutrition in Geriatric Practice: Developing the Short‐Form Mini‐Nutritional Assessment (MNA‐SF),” Journals of Gerontology. Series A, Biological Sciences and Medical Sciences 56, no. 6 (2001): M366–M372, 10.1093/gerona/56.6.M366. 11382797

[20] M. F. Folstein , S. E. Folstein , and P. R. McHugh , ““Mini‐Mental State”: A Practical Method for Grading the Cognitive State of Patients for the Clinician,” Journal of Psychiatric Research 12, no. 3 (1975): 189–198, 10.1016/0022-3956(75)90026-6. 1202204

[21] J. I. Sheikh and J. A. Yesavage , “Geriatric Depression Scale (GDS): Recent Evidence and Development of a Shorter Version,” Clinical Gerontologist 5, no. 1–2 (1986): 165–173.

[22] M. E. Charlson , P. Pompei , K. L. Ales , and C. R. MacKenzie , “A New Method of Classifying Prognostic Comorbidity in Longitudinal Studies: Development and Validation,” Journal of Chronic Diseases 40, no. 5 (1987): 373–383, 10.1016/0021-9681(87)90171-8. 3558716

[23] K. Rockwood , X. Song , C. MacKnight , et al., “A Global Clinical Measure of Fitness and Frailty in Elderly People,” CMAJ 173, no. 5 (2005): 489–495, 10.1503/cmaj.050051. PMC1188185 16129869

[24] H. Komiya , Y. Suzuki , K. Watanabe , et al., “Validation of the Japanese Version of the Clinical Frailty Scale,” Geriatrics & Gerontology International 25, no. 3 (2025): 411–417, 10.1111/ggi.15092. PMC12216798 39895104

[25] S. Y. Park , D. Khemani , A. D. Nelson , D. Eckert , and M. Camilleri , “Rectal Gas Volume Measured by Computerized Tomography Identifies Evacuation Disorders in Patients With Constipation,” Clinical Gastroenterology and Hepatology 15, no. 4 (2017): 543–552.e4, 10.1016/j.cgh.2016.11.013. PMC5362281 27856363

[26] S. Y. Park , D. Khemani , A. Acosta , D. Eckert , and M. Camilleri , “Rectal Gas Volume: Defining Cut‐Offs for Screening for Evacuation Disorders in Patients With Constipation,” Neurogastroenterology and Motility 29, no. 7 (2017): e13044, 10.1111/nmo.13044. PMC5466461 28261935

[27] T. Masaoka , “Current Management of Chronic Constipation in Japan,” Keio Journal of Medicine 72, no. 4 (2023): 95–101, 10.2302/kjm.2022-0036-IR. 37612093

