# Supplementary material for: Association Between CT‐Based Rectal Stool Attenuation and Activities of Daily Living in Hospitalized Older Adults
Source: Geriatr Gerontol Int. 2026 Oct 13;26(10):e70867. doi: 10.1111/ggi.70867 (PMC13659247; doi:10.1111/ggi.70867)
Supplement: Supplementary file 1 [file GGI-26-0-s001.docx]

Supplementary Table 2. Intra-rater reliability of CT-based measurements

| Parameter | ICC | 95% CI |
| --- | --- | --- |
| Stool attenuation | 0.958 | 0.914-0.979 |
| Rectal area | 0.935 | 0.863-0.969 |
| Stool area | 0.961 | 0.920-0.981 |
| Gas area | 0.724 | 0.503-0.857 |

Abbreviations: CI, confidence interval; ICC, intraclass correlation coefficient.
