# Supplementary material for: Association Between CT‐Based Rectal Stool Attenuation and Activities of Daily Living in Hospitalized Older Adults
Source: Geriatr Gerontol Int. 2026 Oct 13;26(10):e70867. doi: 10.1111/ggi.70867 (PMC13659247; doi:10.1111/ggi.70867)
Supplement: Supplementary file 2 [file GGI-26-0-s003.docx]

Supplementary Table 1. Comparison of BADL and IADL According to other CT-based Rectal Parameters

|  | Large rectal area  (>10 cm²; n = 71) | Small  rectal area  (≤10 cm²; n=45) | P value | Large  rectal stool area  (>10 cm²; n = 51) | Small  rectal stool area  (≤10 cm²; n = 65) | P value | Large  rectal gas area  (>10 cm²; n = 13) | Small  rectal gas area  (≤10 cm²; n = 103) | P value |
| --- | --- | --- | --- | --- | --- | --- | --- | --- | --- |
| BADL score, mean (SD) | 67.7 (33.0) | 69.8 (31.3) | 0.734 | 63.2 (33.7) | 72.6 (30.6) | 0.120 | 73.5 (34.2) | 67.9 (32.1) | 0.557 |
| IADL score, mean (SD) | 3.3 (2.8) | 3.6 (3.1) | 0.552 | 2.9 (2.7) | 3.8 (3.0) | 0.080 | 3.6 (2.7) | 3.4 (2.9) | 0.775 |

Abbreviations: BADL, basic activities of daily living; CT, computed tomography; HU, Hounsfield units; IADL, instrumental activities of daily living; SD, standard deviation.
